# Liver X Receptors Protect from Development of Prostatic Intra-Epithelial Neoplasia in Mice

**DOI:** 10.1371/journal.pgen.1003483

**Published:** 2013-05-09

**Authors:** Aurélien J. C. Pommier, Julie Dufour, Georges Alves, Emilie Viennois, Hugues De Boussac, Amalia Trousson, David H. Volle, Françoise Caira, Pierre Val, Philippe Arnaud, Jean-Marc A. Lobaccaro, Silvère Baron

**Affiliations:** 1 Clermont Université, Université Blaise Pascal, Génétique Reproduction et Développement, BP 10448, Clermont-Ferrand, France; 2 CNRS, UMR 6293, GReD, Aubiere, France; 3 INSERM, UMR 1103, GReD, Aubiere, France; 4 Centre de Recherche en Nutrition Humaine d'Auvergne, Clermont-Ferrand, France; University of Washington, United States of America

## Abstract

LXR (Liver X Receptors) act as “sensor” proteins that regulate cholesterol uptake, storage, and efflux. LXR signaling is known to influence proliferation of different cell types including human prostatic carcinoma (PCa) cell lines. This study shows that deletion of LXR in mouse fed a high-cholesterol diet recapitulates initial steps of PCa development. Elevation of circulating cholesterol in *Lxrαβ-/-* double knockout mice results in aberrant cholesterol ester accumulation and prostatic intra-epithelial neoplasia. This phenotype is linked to increased expression of the histone methyl transferase EZH2 (Enhancer of Zeste Homolog 2), which results in the down-regulation of the tumor suppressors *Msmb* and *Nkx3.1* through increased methylation of lysine 27 of histone H3 (H3K27) on their promoter regions. Altogether, our data provide a novel link between LXR, cholesterol homeostasis, and epigenetic control of tumor suppressor gene expression.

## Author Summary

Cholesterol is one of the major metabolic molecules required for a broad range of cellular processes. Recent advances in prostate cancer research have demonstrated that tumor cells need to increase their supply of cholesterol to sustain membrane building, proliferation, and survival capacities. Liver X receptors, which belong to the nuclear receptor superfamily, are central mediators of cholesterol homeostasis. Indeed, they regulate the expression of many genes involved in cholesterol uptake storage and efflux. Here, we show that genetic ablation of LXRs in mice results in the formation of precancerous lesions in the prostate, called prostatic intra-epithelial neoplasia. These are only observed when mice are fed a high-cholesterol diet. Hence, LXRs regulate cholesterol homeostasis in the prostate and protect cells from abnormal proliferation when exposed to high dietary cholesterol.

## Introduction

The Liver X Receptors (LXRα, encoded by the gene *Nr1h3*, and LXRβ, encoded by the gene *Nr1h2*) belong to the nuclear receptor superfamily and bind to naturally occurring oxidized forms of cholesterol, known as oxysterols [1]–[3]. These receptors heterodimerize with RXR (Retinoid X Receptor) and stimulate various target genes expression, among which, genes encoding proteins in charge of cholesterol efflux, storage and uptake. Deletion of these receptors in mouse has been previously associated with the development of benign prostatic hyperplasia (BPH) lesions in ventral prostates [4], [5]. These findings enlighten the role of LXR in prostate homeostasis. However, BPH and prostate cancer (PCa) appear in distinct regions of the prostate and have distinct etiologies. Therefore, not much is known about PCa and LXR *in vivo*. Consistent with a potential role in prostate tumor formation, LXR have been reported to modulate proliferation [6], [7] and survival [8] of human prostatic cells in culture and in xenograft models. In these models, inhibition of proliferation through LXR activation was inversely correlated with expression of the ATP-binding cassette A1 (*ABCA1*) and G1 (*ABCG1*), two known target genes of LXR, which are involved in cholesterol efflux [9]. These observations suggest that the tumor suppressive activity of LXR on human PCa cell lines could result from their capacity to limit intracellular cholesterol concentration. This notion was supported *in vivo* by exposure of the *tr*ansgenic *a*denocarcinoma of the *m*ouse *p*rostate (TRAMP) model, which carries a transgene encoding the SV40 large T antigen driven by the probasin promoter, to a high cholesterol diet. In TRAMP mice, this diet led to an acceleration of prostate tumor development [10]. A similar diet also increased aggressiveness of tumors generated by LNCaP cells in xenograft experiments [11]. On the basis of these observations, we hypothesized that LXR, through control of cholesterol metabolism, could act as “gatekeeper” preventing prostate tumor development. Thus we investigated the consequence of LXR ablation in the dorsal prostates of mice fed a high cholesterol diet.

## Results

### Development of Prostatic Intra-Epithelial Neoplasia in Prostates of LXR Knockout Mice Fed a High-Cholesterol Diet

**Figure 1:**
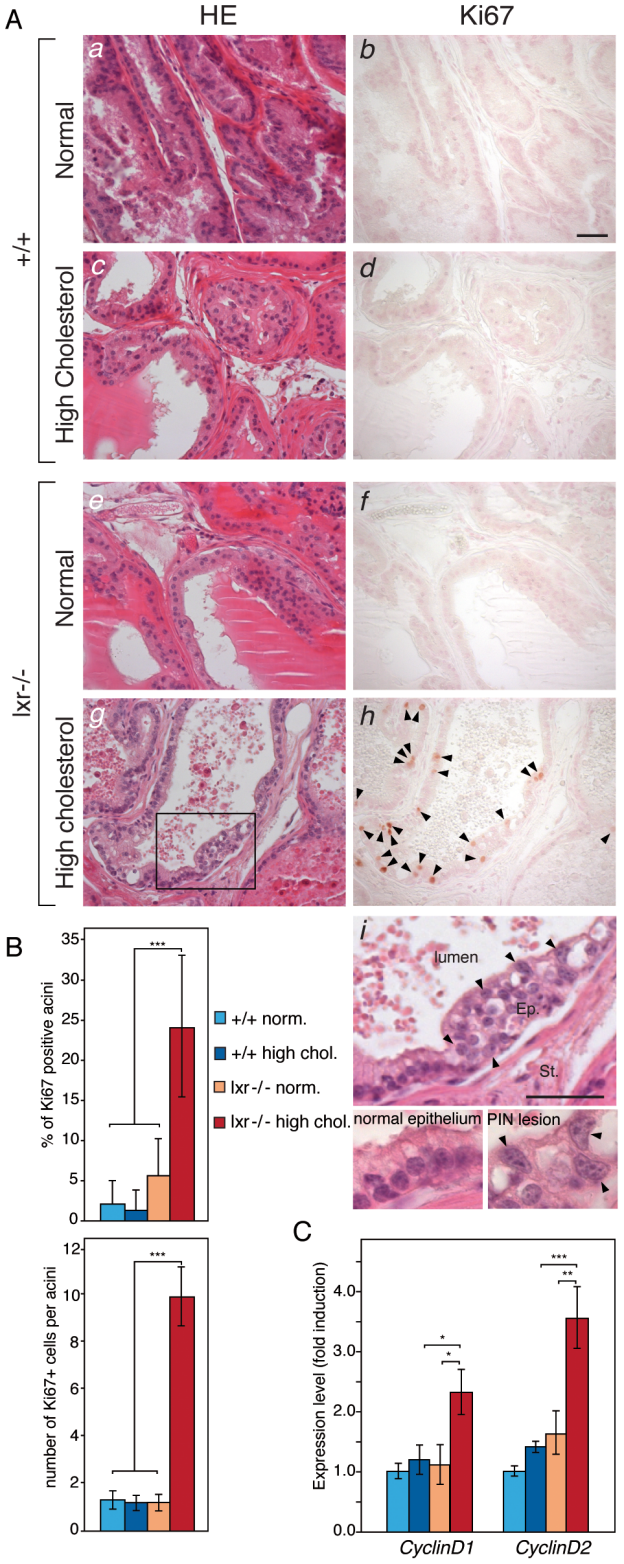
High-cholesterol diet induces proliferation in LXR mutant mouse prostate. (A) Histological sections of dorsal prostate lobes of 5 month-old WT (*a,b,c,d*) and LXR null mice (*e,f,g,h*) fed normal or high cholesterol diet were analyzed after H&E staining (*Left*) or Ki67 IHC (*Right*). Arrowheads point Ki67-positive cells. Higher magnification of the prostatic epithelium of LXR null mice fed a high cholesterol diet revealed abnormal features (*i*). Arrowheads indicate atypical cells with enlarged nuclei and prominent nucleoli which represent typical signs of PIN. Ep: Epithelium, St: Stroma (Scale bars = 50 µm). (B) IHC for Ki67 was quantified by counting the percentage of prostatic acini with proliferative cells and the average Ki67+ cell number in proliferative acini (N = 6 per group). (C) qPCR analysis of *CyclinD1* and *CyclinD2* expression (N = 9/13 per group). * p<0.05, ** p<0.01, *** p<0.001 in Student's *t* test. Error bars represent the ± mean SEM.

Under a standard diet, dorsolateral prostates of *Lxrαβ-/-* double knockout mice (*Lxr-/-*) were histologically indistinguishable from their wild-type (WT) counterparts, as shown by H&E staining (Figure 1A*a* and *e*) and Ki67 IHC (Figure 1A*b* and *f*). In order to increase circulating cholesterol levels, WT and knockout mice were fed a standard or a hypercholesterolemic diet, as previously described [11], [12]. This cholesterol surge had no effect on the gross histology of WT dorsolateral prostates (Figure 1A*c*). In contrast, analysis of LXR mutant prostates revealed a disorganization of the epithelial layer, which was reminiscent of PIN grade II [13] (Figure 1A*g*), characterized by the formation of cribriform and tufting patterns. Nuclei were enlarged and displayed prominent nucleoli (Figure 1A*i*). The PIN status of the lesions was confirmed by an increased proliferation as demonstrated by Ki67 staining (Figure 1A*h*, 1B) and *Cyclin D1* and *D2* overexpression (Figure 1C). The PIN phenotype was restricted to the dorsolateral prostate (Figure S1A, S1B) and was dependent on the ablation of both *Lxrα* and *Lxrβ*. Indeed, single knockout prostates were comparable with WT glands in terms of histology and proliferation (Figure S1C, S1D).

### Increased Turnover of Epithelial Cells in LXR Mutant Mice under High-Cholesterol Condition

**Figure 2:**
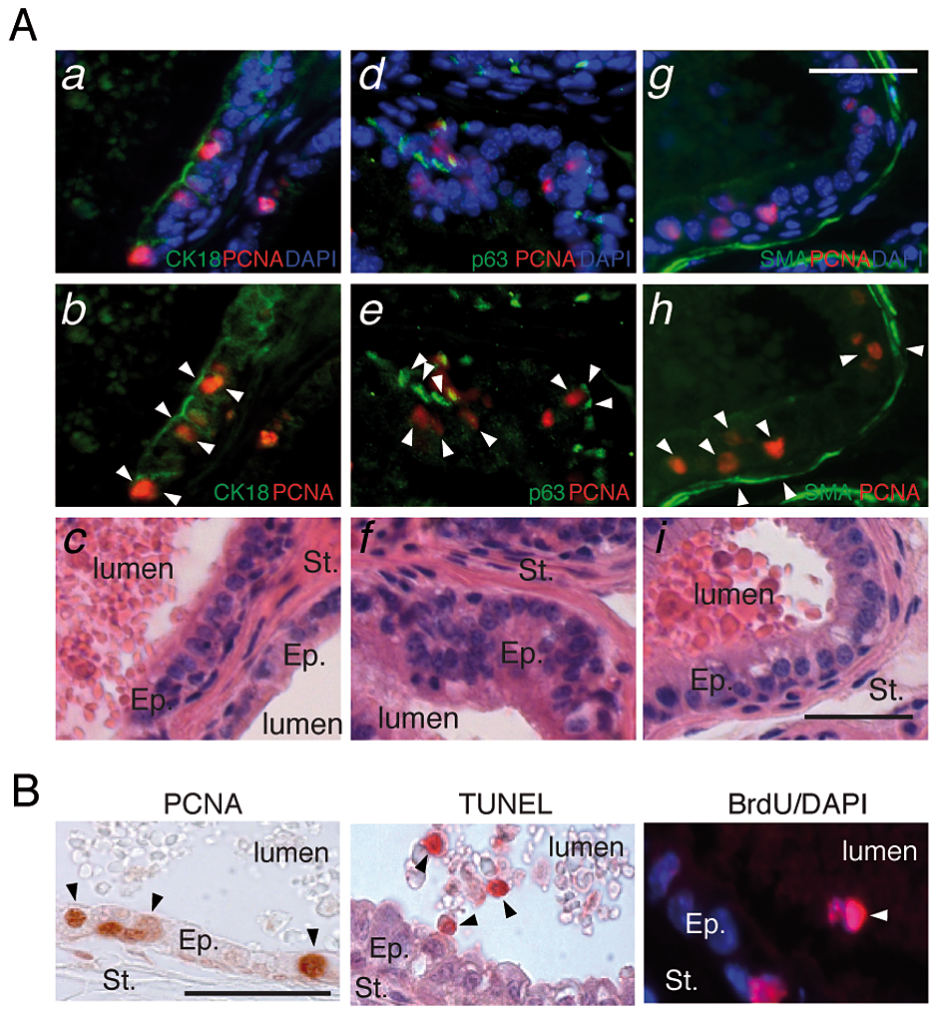
LXR null mice exhibit aberrant epithelial cell renewal. (A) Proliferative cells in LXR knockout prostates under high cholesterol condition were identified by H&E staining and double-IHC with antibodies directed against PCNA and specific markers for luminal epithelial cells (CK18) (*a,b,c*), basal cells (p63) (*d,e,f*) and smooth muscle (SMA - smooth muscle actin) (*g,h,i*). Ep: Epithelium, St: Stroma (Scale bar = 10 µm). (B) PCNA immunodetection (proliferation), TUNEL staining (apoptotic nuclei) and BrdU immunodetection (cumulative proliferation) were performed on dorsal prostates of LXR null mice under high cholesterol condition (Scale bar = 10 µm). Arrowheads point to regions of interest.

The identity of proliferative cells was determined by immunofluorescence analyses using markers for prostatic cells subtypes. To identify proliferative cells within the different prostatic compartments, we performed double staining for PCNA and CK18 (luminal cells), p63 (basal cells) or SMA (stromal smooth muscle cells). Most PCNA+ cells were positive for CK18 (Figure 2A*a*, *b*, and *c*) and were surrounded by p63+ epithelial basal cells (Figure 2A*d*, *e* and *f*). Occasionally, p63+;PCNA+ cells were observed (data not shown), indicating that all the epithelial lineage could be targeted by proliferation in LXR null mice fed a high cholesterol diet. PCNA+ cells were exclusively localized inside the epithelium delineated by smooth muscle actin (SMA) staining (Figure 2A*g*, *h* and *i*). PCNA+ or Ki67+ cells were not observed in the stroma (data not shown). Altogether, these results indicated that proliferation was restricted to the epithelial compartment. This was consistent with previous observations in the ventral prostate lobes of LXR mutant mice [4]. Presence of abnormal proliferation in the epithelium suggested that cell renewal could be deregulated. TUNEL staining showed increased apoptosis in the epithelium (Figure S2A, S2B) and identified delaminating apoptotic cells inside the lumen (Figure 2B). BrdU+ cells were also present inside prostatic ducts, suggesting that proliferative cells could detach into the lumen (Figure 2B). The increase of apoptosis could be the result from cholesterol cytotoxicity as shown in cholesterol-overloaded foam cells in atherosclerosis [14]. However, a similar cell death surge has been reported in a PTEN-deficient mouse prostates [15], [16]. In prostate of *Lxr-/-* mice under high cholesterol condition, it could therefore be a consequence of pathological development. Altogether, these observations suggested that the epithelium of LXR null mice presented both increased proliferation and apoptosis that resulted in an alteration of cell turnover.

### Cholesterol Metabolism Is Altered in LXR Knockout Mouse Prostate Fed a High-Cholesterol Diet

**Figure 3:**
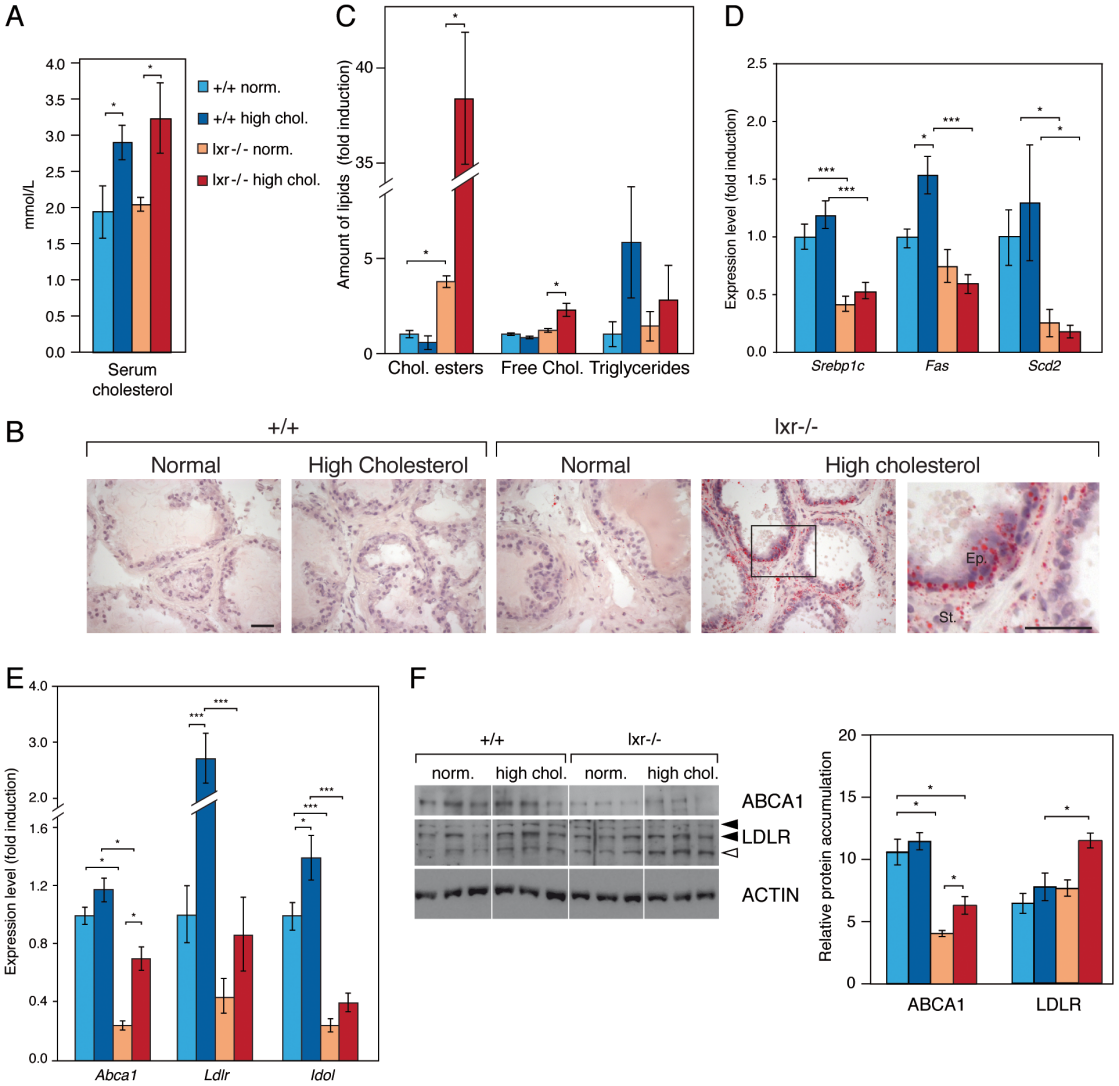
Prostates of LXR mutant mice accumulate cholesterol esters through inappropriate LXR target genes regulation. (A) Plasma concentrations of cholesterol were determined (N = 9/13 per group) after 5 weeks dietary conditional exposure in each genotype. (B) Neutral lipids accumulation was observed after Oil-Red-O staining (ORO) (Scale bars = 50 µm). (C) Cholesterol esters, free cholesterol and triglycerides were quantified by thin layer chromatography (N = 3 per group). (D) *Srebp1c*, *Fas* and *Scd2*, (E) *Abca1*, *Ldlr* and *Idol* transcript levels were determined by qPCR (N = 9/13 per group). (F) Total protein lysates of WT and LXR null mice under normal or high cholesterol diet were analyzed by western blotting with antibodies against ABCA1, LDLR and ACTIN as a loading control (left panel), quantification of ABCA1 and LDLR protein accumulation levels (right panel). * p<0.05, *** p<0.001 in Student's *t* test. Error bars represent the ± mean SEM.

LXR are essential regulators of lipid metabolism. However, there was no major difference in circulating cholesterol levels in LXR knockout mice when compared with WT, irrespective of the diet (Figure 3A). Therefore, we speculated that the PIN phenotype resulted from deregulated lipid metabolism within the prostate. Indeed LXR knockout prostates accumulated large amounts of Oil-Red-O staining under high cholesterol condition, consistent with neutral lipid accumulation (Figure 3B). Quantitative analyses revealed a significant accumulation of cholesterol esters in LXR mutant mice fed a standard diet, which was largely amplified when mice were fed the hypercholesterolemic diet (Figure 3C). This phenotype was also associated with an increase in free cholesterol. Intra-prostatic triglycerides concentration was not altered and expression of genes involved in lipogenesis was even inhibited in LXR knockout prostates compared with WT (Figure 3C, 3D). This suggested that the accumulation of neutral lipids in the prostate of LXR knockout mice resulted from a deregulation of cholesterol transport in prostatic cells. Indeed, expression of *Abca1*, the transporter in charge of cholesterol efflux, was decreased both at the mRNA and protein levels in LXR knockout prostates (Figure 3E, 3F). Conversely, LDLR protein accumulation was increased by LXR ablation (Figure 3F, white arrow), even though *Ldlr* mRNA accumulation was decreased (Figure 3E). This was correlated with a decreased expression of the LXR target gene *Idol* (Figure 3E), which catalyzes the ubiquitination and subsequent degradation of LDLR [17]. Therefore, aberrant cholesterol ester accumulation in LXR deficient prostatic cells results from both increased uptake and decreased efflux.

### Prostatic Gene Expression Signature of LXR Mutant Mice Fed a High-Cholesterol Diet

**Figure 4:**
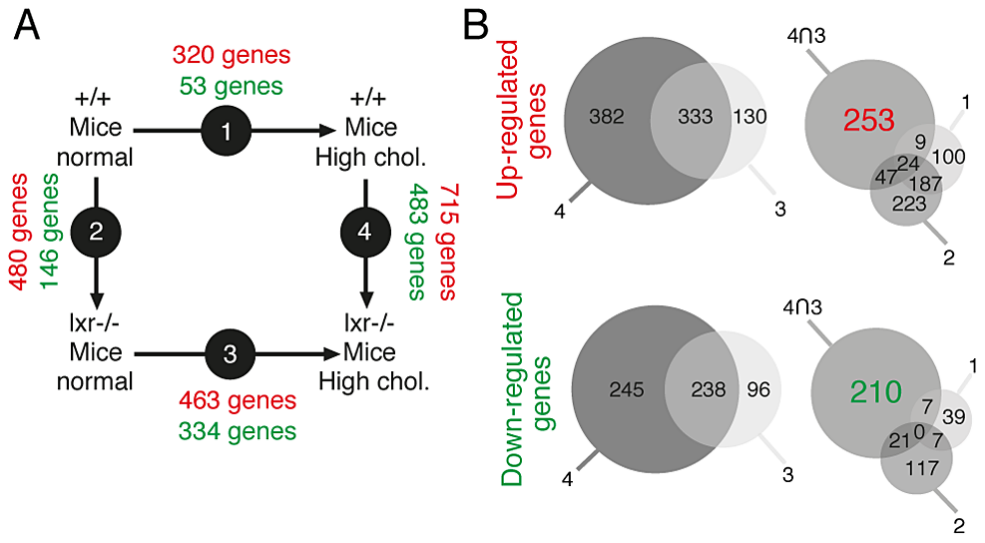
Identification of genes associated with the occurence of PIN lesions. ****(A) Experimental design of gene expression profiling studies. (B) Venn diagram analysis was used to isolate genes associated with PIN development in LXR null mice under high cholesterol diet: genes deregulated in both arrays 3 and 4 were selected and genes deregulated in arrays 1 and 2 were further subtracted from this list. This method leads to the extraction of 463 genes (253 up- and 210 down-regulated).

**Figure 5:**
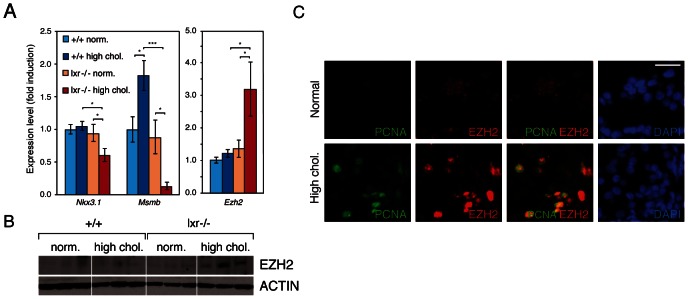
Disruption of cholesterol homeostasis induces the repression of *Nkx3.1* and *Msmb* tumor suppressor genes and upregulation of the *Ezh2* histone methyltransferase gene. (A) *Nkx3.1*, *Msmb* and *Ezh2* expression levels were analyzed by qPCR (N = 9/13 per group). (B) Western blot analysis of EZH2 accumulation in total protein lysates from dorsal prostate of WT and LXR null mice under normal or high cholesterol diet. (C) Immunofluorescence analyses were carried out on LXR null mice under normal or high cholesterol diet using antibodies directed against PCNA and EZH2 (Scale bar = 5 µm). * p<0.05, *** p<0.001 in Student's *t* test. Error bars represent the ± mean SEM.

**Figure 6:**
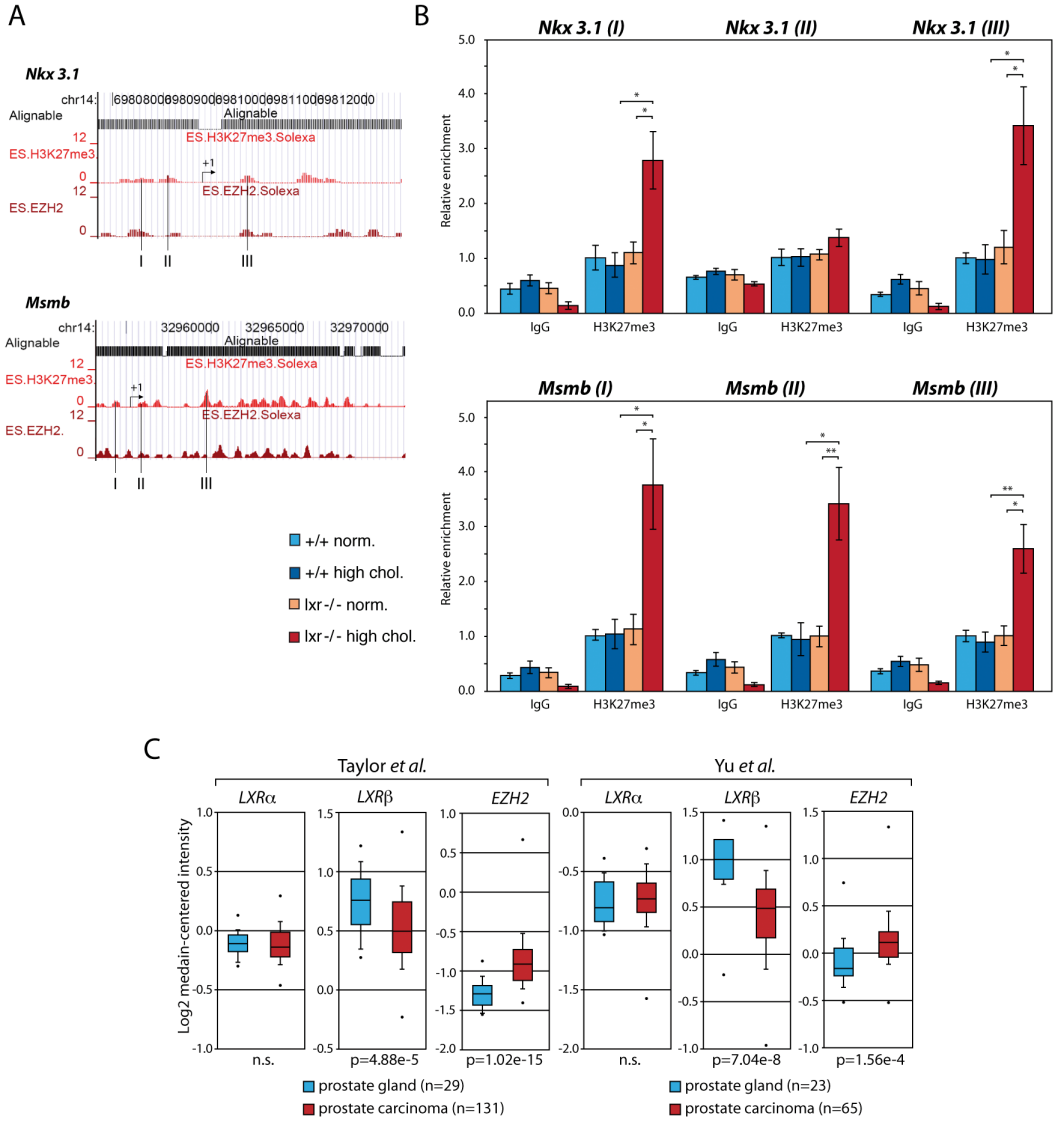
Upregulation of *Ezh2* leads to increased enrichment of the H3K27me3 histone mark on *Nkx3.1* and *Msmb* promoter regions. (A) Location of loci I, II and III amplified by qPCR on H3K27me3 mark profiles and Ezh2 occupancy sites on *Nkx3.1* and *Msmb* promoters as identified by ChIP-seq in ES cells [45] (http://www.broadinstitute.org/scientific-community/science/programs/epigenomics/chip-seq-data). (B) ChIP analyses using antibodies raised against trimethylated H3K27 *vs.* negative control IgG (N = 3/6 per group). Histograms show relative enrichment values of Loci I, II and III (bound/input) on chromatin obtained from WT and LXR null mice under normal or high cholesterol diet. (C) Oncomine boxed plot analysis (http://www.oncomine.org) of *LXRα, LXRβ* and *EZH2* expression levels between healthy prostate glands and human PCa in datasets referenced in [21] and [20] (n.s.; non-significant). * p<0.05, ** p<0.01 in Student's *t* test. Error bars represent the ± mean SEM.

Our data showed that control of cholesterol homeostasis by LXR is crucial to restrain epithelial cell proliferation in the prostate. In order to determine key molecular events resulting from elevation of cholesterol in the prostate, we designed microarray experiments. We compared prostatic gene expression of WT and LXR mutant mice in normal and high dietary cholesterol conditions (Figure 4A). The list of up- and down-regulated genes has been established on the basis of signal intensity, Log ratio and *p*-value (Figure S3). The highest number of deregulated genes was observed when WT and LXR knockout mice were exposed to high circulating cholesterol levels, again emphasizing the central role of cholesterol in the establishment of the phenotype (Figure 4A). In order to determine gene expression signature of the PIN phenotype in LXR mutant mice fed a high cholesterol diet and to identify relevant molecular events, we have restricted the gene list using Venn analysis. We selected common deregulated genes associated with the PIN phenotype and eliminated those that were sensitive to diet and/or LXR ablation alone. Therefore, we focused on the genes involved in the establishment of the PIN phenotype by selecting genes that were deregulated in both arrays 3 (lxr-/- normal *vs.* lxr-/- high chol.) and 4 (+/+ high chol. *vs.* lxr-/- high chol.) and by subtracting genes that were deregulated in both arrays 1 (+/+ normal *vs.* +/+ high chol.) and 2 (lxr-/- normal *vs.* +/+ normal). This resulted in a list of 463 genes (Dataset S1), 253 up and 210 down (Figure 4B). Ingenuity Pathway Analysis (IPA) was used to investigate potential biological processes that underlay the PIN phenotype of LXR mutant mice (Figure S4). The second most significantly enriched gene-category was ‘cancer’, which was associated with a large list of 146 genes (Dataset S2). More than 50% of these 146 genes were also deregulated in a mouse model of prostate cancer resulting from PTEN deletion in prostatic epithelium [18] (data not shown). This strongly suggested that the PIN lesions observed in LXR knockout mice in the high cholesterol condition were genuine pre-cancerous alterations. Interestingly, this analysis showed down-regulation of two well described prostatic tumor suppressor genes *Nkx3.1* and *Msmb* (Dataset S2, highlighted in red), which was further confirmed by qPCR analysis (Figure 5A, Figure S5). These two genes were specifically found in gene categories such as tumor development, cell proliferation and prostate organogenesis (Dataset S3, highlighted in red). *Nkx3.1* and *Msmb* promoters have recently been demonstrated to be targets of the histone methyl transferase EZH2 that represses gene expression through H3K27 trimethylation. qPCR and western blot analyses showed that *Ezh2* was specifically overexpressed in LXR knockout prostates when animals were fed a high cholesterol diet (Figure 5A, 5B). Immunohistochemistry further confirmed overaccumulation of EZH2 in proliferative PCNA+ cells in LXR knockout prostates, when animals fed a high cholesterol condition (Figure 5C). This suggested that the effect of cholesterol on the development of PIN was dependent on down-regulation of *Nkx3.1* and *Msmb*, resulting from EZH2-mediated modification of their promoter chromatin. Indeed, ChIP analyses confirmed that nucleosomes at both *Nkx3.1* and *Msmb* promoters were significantly trimethylated on H3K27 in the prostates of LXR null-mice fed a high cholesterol diet (Figure 6A, 6B). Interestingly, *Msmb* expression was increased by a high cholesterol diet in WT mice. This was independent of *Ezh2*, whose expression was unaltered (Figure 5A). Such observation indicates that other mechanisms are involved in the regulation of this tumor suppressor gene expression and that it is highly sensitive to metabolic changes in prostate tissue. To further confirm the potential link between *LXR* and *EZH2* expression, we performed a retrospective study of publicly available DNA microarray data of human PCa cohorts, using Oncomine. These analyses showed that *LXR*β expression was significantly down-regulated in prostate carcinomas compared to normal tissue and that this down-regulation was associated with increased *EZH2* expression (Figure 6C). Interestingly, careful analysis of normal prostate gland as well as metastasis heat maps revealed that levels of *LXR*β, *EZH2* and *MSMB* were tightly coordinated between each other (Figure S8). The expression pattern of *NKX3.1* present no significant modification. Therefore, the connection between LXR, cholesterol homeostasis, *EZH2* and *MSMB* expression that we uncovered in mouse could also be relevant in human PCa.

## Discussion

Previous analyses of LXR null mice have shown the development of a BPH-like phenotype in the ventral lobe of the prostate [4], [5]. However in patients, BPH arises in the periurethral and transition zones distinct from the peripheral zone from which cancer emerges. Therefore, to date, the role of LXR in PCa had been postulated on the basis of studies performed in tumor cell lines [6]–[9]. Here we show for the first time that LXR ablation results in the development of PIN in the dorsal prostate in mouse, which is the most similar lobe to human peripheral prostate, the area from which the majority of cancerous lesions occurs in human [18]. Consistent with previously published data [4], this phenotype is not observed under normal dietary conditions. Indeed, in our model, PIN development is associated with a high cholesterol diet, which results in prominent intra-prostatic accumulation of cholesterol ester. Cholesterol has been extensively associated with prostate malignancy [19]. We therefore hypothesize that increased cholesterol ester storage is a major contributor to the appearance of the PIN phenotype. Interestingly, abnormal cholesterol storage was also observed in LXR mutant mice fed a standard diet, albeit to a lesser extent. Absence of PIN development under this condition, even in 18 month-old animals (data not shown) suggests that cholesterol accumulation needs to be tipped over a threshold to become deleterious. It is therefore tempting to speculate that in patients, the combination of metabolic disease and/or high cholesterol diet with abnormal LXR activity may favor prostate cancer development, by increasing cholesterol accumulation beyond this threshold. Consistent with this idea, we show decreased expression of *LXRβ* in prostatic carcinomas compared with normal prostate (Figure 6C) [20], [21]. Numerous *in vivo* and *ex vivo* studies have shown the sensitivity of already transformed tumor cells to variations in cholesterol supply and *de novo* synthesis [8], [11], [19], [22]. Our data goes one step further by showing that LXR ablation and the subsequent accumulation of cholesterol may in fact initiate neoplastic development in the prostate.

The molecular mechanism by which LXR control cell cycle in human prostatic tumor cell lines is still poorly understood. LXR activation has been shown to slow down the cell cycle through accumulation of the p27 cell cycle inhibitor and downregulation of SKP2 in LNCaP cells [6]. RNA interference demonstrated that part of this antiproliferative effect was supported by LXR themselves [23]. Interestingly, aberrant proliferation observed in LXR null mice fed a high cholesterol diet was found in only 24% of the acini (Figure 1B). These findings indicate that the cellular context of one particular epithelial cell plays an essential role in cell cycle deregulation and in the development of PIN lesions. It is therefore very likely that the prostatic phenotype of LXR-null mice is not only dependent on an epithelial cell-autonomous effect of LXR ablation. This hypothesis is supported by our previous observation that LXR were required to establish a cellular dialogue between stromal and epithelial compartments in ventral prostate [5].

One interesting observation of our study is the correlation between increased cholesterol accumulation and increased expression of *Ezh2*. Overexpression of *EZH2* is associated with aggressive prostate carcinomas in patients [24] and has been shown to control prostate cell proliferation through epigenetic silencing of the tumor suppressors *NKX3.1* and *MSMB* [25], [26]. Here, we show that the combination of LXR ablation and high cholesterol diet is associated with decreased *Nkx3.1* and *Msmb* expression, which is correlated with an increase in the H3K27me3 mark on their promoter regions. It is therefore tempting to speculate that some of the oncogenic effects of cholesterol accumulation in the context of LXR ablation are mediated by up-regulation of EZH2 and the conscutive gene silencing. How this is achieved is still unclear. However two scenarios could account for such a mechanism. In the first scenario, deregulation of *Ezh2* expression could be triggered in an epithelial cell-autonomous fashion as lipids (PUFA) have been already identified in such a process [27]. However, the underlying molecular mechanisms remain unknown as the promoter sequences of *Ezh2* are still poorly characterized [25]. In the second scenario, *Ezh2* overexpression could result from an accumulation of a specific epithelial cell compartment. EZH2 is not a canonical stem/progenitor marker in the prostate but has been involved in cancer stem cell maintenance in various diseases [28], [29]. In human prostate, a minor subgroup of “stem” cells (CD44+, Oct4+) expresses *EZH2* and has been proposed to represent a cell reservoir for prostatic adenocarcinoma initiation [30]. Consequently, increased expression of *Ezh2* in LXR null mice could result from expansion of a progenitor epithelial cell population. The effect of LXR ablation and cholesterol accumulation on epigenetic processes is likely to extend beyond EZH2. Indeed, we show increased expression of *Uhrf1* in correlation with *Ezh2* accumulation in LXR mutant mouse prostates, under high cholesterol condition (Figure S6). This is consistent with reports of a positive correlation between these two factors in human prostate tumors. UHRF1 acts with Suv39H1 and DNA methyltransferases to alter histone H3K9 methylation, acetylation and DNA methylation to epigenetically repress target genes. Furthermore, UHRF1 and EZH2 have been proposed to synergistically promote inactivation of oncosuppressor genes, among which *Nkx3.1* and *Msmb* [31], in tumor cells. Consistent with the idea that *Ezh2* deregulation results from interactions between different cell compartments of the prostate and thus from expansion of Ezh2-positive cells, LXR activation or knockdown did not change EZH2 accumulation in prostatic culture cell lines (data not shown). Another intriguing observation regards the upregulation of *Msmb* in WT mouse prostate under high cholesterol condition (Figure 5A). Transcriptional regulation of *Msmb* is poorly characterized beyond the role of EZH2 and androgens [26], [32]. Since levels of androgen target genes, as Nkx3.1 [33], [34], were unchanged (data not shown), we hypothesized that androgen amount was stable irrespective of the diet. Thus we concluded that upregulation of *Msmb* expression was not due to a higher level of androgens. It was also unlikely be dependent on EZH2, whose expression was unaltered in response to cholesterol in WT mouse prostate (Figure 5). Taken together, these observations suggest that *Msmb* is sensitive to prostate metabolic status and that an unknown mechanism yet is involved. Given the role of *Msmb* repression as a maker of prostate cancer progression and a *bona fide* tumor suppressor gene [35]–[37], we speculate that *Msmb* overexpression in WT mice prostates represents a defensive molecular mechanism against the metabolic stress induced by a high cholesterol diet.

Among canonical LXR functions, *primum movens* leading to PIN phenotype in prostate of *Lxr*-null mice could originate from deregulation of inflammatory response in prostate tissue as suggested by gene ontology (Dataset S3). Indeed, inflammation has been widely associated with prostate cancer development. Even though there was no clear CD45+ staining *Lxr*-/- in dorsal prostate in high cholesterol condition (Figure S7A), *Cd45* expression measured by qPCR was 2-fold increased compared to WT (Figure S7B). Moreover, analysis by hierarchical clustering comparing array 1 and array 4 of inflammation-associated genes expressions (Figure S7C) showed that mouse prostate displayed a specific gene signature. While a high cholesterol diet in prostate of WT mice induces expression of inflammatory genes without leading to an *in vivo* phenotype, some of these genes failed to be upregulated in LXR mutant mice (Figure S7C, compared group 1 and 2). Conversely, genes that were insensitive to a high cholesterol diet in WT mice, showed a massive deregulation in LXR mutant mice in similar diet conditions (Figure S7C, group 3). Altogether, prostate of LXR mutant mice exhibits a specific gene expression signature that revealed a deregulation of the inflammatory network. This raises the question of LXR-dependent regulation of inflammation in prostate tissue and its impact on the PIN development.

Human dataset analysis pointed out that *LXRβ* but not *LXRα* expression could be linked to *EZH2* expression while both isoforms need to be invalidated to induce a PIN occurrence in mice (Figure S8). Absence of any change in LXRα expression could explain the lack of a clear deregulation of some LXR target genes in Oncomine datasets (data not shown). Moreover, both LXRα and LXRβ have been demonstrated to be expressed and functional in human PCa cells [8], [38]. These observations suggest that *EZH2* deregulation could be linked to a mechanism specifically depending on LXRβ. Such specificity has already been shown in human, particularly in a study on preeclampsia providing a LXRβ-dependent risk in this pathology [39]. Another point emphasized by the human dataset is the absence of *NKX3.1* expression changes between normal prostate, carcinoma and metastasis group in both examined cohorts (Figure S8). *NKX3.1* expression profiles are somehow unexpected, as this gene has been largely reported as a tumor suppressor gene in the prostate. Nevertheless, various mechanisms have been demonstrated to repress *NKX3.1* during carcinogenesis and these observations suggest that filtrating analysis of human datasets based on association with identified oncogenic alterations, such as *PTEN* inactivation [40], should me more informative. Altogether, our results show that LXR act as “gate keeper” in mouse prostate to prevent cholesterol accumulation and subsequent PIN development. Our findings further suggest that the metabolic status of the prostate can govern epigenetic processes involved in prostate cancer progression.

## Methods

### Animals

*Lxrα* and *lxrβ* double knockout mice and their wild-type controls [41], [42], [43] were maintained on a mixed strain background (C57BL/6:129Sv) and housed according to local ethical regulations. Mice were fed *ad libitum* a normal mouse chow (Global-diet 2016S) until 5 months of age. Mice were then fed either a normal or hypercholesterolemic diet (Teklad diet number 88051; Harlan, Gannat, France) for 5 weeks. Animals were sacrificed, blood plasma was collected and prostates were dissected. For histological analysis, prostates were either embedded in NEG 50 (Thermo Scientific, Kalamagoo, MI, USA) or fixed in an alcohol/formaldehyde 37% and acetic acid mixture (7.5∶2∶0.5; v/v) before embedding in paraffin for histological analysis. For lipid, protein and RNA extractions, prostates were snap-frozen in liquid nitrogen. All animals were maintained in a controlled environment and animal care was conducted in compliance with the national standards and policies (C 63 014.19). The Regional Ethics Committee approved all experiments (CE 74-12 S) (Text S1).

### Staining, Immunohistochemistry, Immunofluorescence, and TUNEL

Prostate tissues were fixed overnight in 4% paraformaldehyde, paraffin-embedded, sectioned and stained with hematoxylin and eosin according to a standard protocol. For immunochemistry, paraffin sections were dewaxed, rehydrated, unmasked using 0.1M citrate buffer (pH 6.0) and then incubated with primary antibodies overnight at 4°C in a humidified chamber. Primary antibodies were: PCNA (FL-261) sc-7907 (Santa Cruz Biotechnology, Santa Cruz, CA), EZH2 (AC22) #3147 (Cell signaling, Montigny-Le-Bretonneux, France), BrdU (Roche diagnostic, Meylan, France), p63:69241A (BD Pharmigen, San Diego, CA, USA), Cytokeratin 18 (H-80) sc-28264 (Santa Cruz Biotechnology, Santa Cruz, CA), Actin A2066 (Sigma-Aldrich). Detections were performed alternatively using the NovaRED substrate kit for peroxidase (Vector Laboratories, Burlingame, CA) or Alexa 488 conjugated anti-mouse IgG/Alexa 555 conjugated anti-rabbit IgG (Invitrogen). Cell nuclei were stained using Hoechst 33342 (Sigma-Aldrich) at 1 mg/ml.

Apoptotic nuclei were visualized through a TUNEL reaction relying on terminal deoxynucleotidyl transferase (TdT; Euromedex, Souffelwegersheim, France) and biotin-11-dUTP (Euromedex), dATP (Promega, Charbonnière, France). Positive nuclei were revealed by addition of extravidin-coupled alkaline phosphatase and FastRed TR/Naphthol AS-MX substrate (Sigma-Aldrich). Nuclei were counterstained with Mayer hematoxylin solution. Cross-sectional areas of the prostate were photographed using a Zeiss Axioplan fluorescence microscope and the Axiovision 4.2 software (Carl Zeiss Vision GmbH, Le Pecq, France). Lipid stainings were performed on cryosections with Oil-Red-O (Sigma-Aldrich) as previously described [44].

### Transcriptomic and Pathway Analyses

Microarray study is detailed in Text S1. Briefly, mRNA samples were analyzed using Agilent 44K Whole Mouse Genome microarrays (Agilent Technologies, Palo Alto, CA). For each microarray, log ratio, fold-change and *p*-value were determined using the Rosetta Resolver Gene Expression Analysis System and these criteria were used for Venn analysis by threshold method. Microarrays results were deposited in the EBI MIAME-compliant database (E-MTAB-546).

### Real-Time PCR

Total RNAs were isolated using NucleoSpin RNA II column kit (Macherey-Nagel, Hoerd, France). cDNAs were synthesized with Moloney Murine Leukemia Virus Reverse Transcriptase (Promega) and random hexamer primers (Promega) according to the manufacturer's instructions. cDNA templates were amplified by MESA GREEN MasterMix Plus for SYBR Assay (Eurogentec, Seraing, Belgium) using an iCycler (Bio-Rad, Marnes-la-Coquette, France). Primer sequences are listed in Text S1. qPCR results were normalized alternatively using *36b4* or *18S* as a standard.

### Lipids

Blood concentrations of circulating cholesterol were determined on an automated clinical chemistry analyzer (Roche Diagnostics) according to manufacturer's instructions. Lipid samples from prostate tissues were extracted by the *Folch* method as previously described [8] and analyzed on high-performance thin layer chromatography (TLC) plates.

### Western Blot

Proteins were extracted in Hepes 20 mM, NaCl 0.42 M, MgCl_2_ 1.5 mM, EDTA 0.2 mM and NP40 1% supplemented with PMSF 1 mM (Sigma-Aldrich), Complete 1X (Roche Molecular Biochemicals, Meylan, France), NaF 0.1 mM and Na_2_VO_3_ 0.1 mM (Sigma-Aldrich). For western blot, 40 µg of protein lysates were separated by SDS PAGE and were incubated with antibodies against Actin A2066 (Sigma-Aldrich), ABCA1 NB400-105 (Novus, Littletown, CO), EZH2 (AC22) #3147 (Cell Signaling) and LDLR 10007665 (Cayman Chemical).

### Chromatin Immunoprecipitation

Chromatin preparation from dorsolateral prostate and for immunoprecipitation has been described previously (3). Immunoprecipitation was performed using Anti-trimethyl Histone H3 (Lys27) #ABE44 (Millipore, Billerica, MA) and negative control IgG #Kch-504-250 (Diagenode, Liège, Belgium). Primers used for qPCR analysis are listed in Text S1.

### Statistics

qPCR data, lipids assays and Ki67-staining parameters are expressed as mean ± standard deviation. Statistical analysis was performed with a two-tailed Student's *t* test.

## Supporting Information

Dataset S1 List of 463 Genes Identified Using Venn Analysis.
(XLSX)

Dataset S2 List of 146 “Cancer” Genes Identified Using Ingenuity Pathway Analysis.
(XLSX)

Dataset S3 Table of Gene Categories Enrichment of the 463 Genes Unveiled by Venn Analysis Using Ingenuity Pathway Analysis.
(XLS)

Figure S1 Analysis of Cell Proliferation in *Lxr*α, *Lxr*β Single Knockout Mice and Weights of Prostatic Lobes. (A) *CyclinD1* expression levels were analyzed by qPCR (N = 9/13 per group) in ventral (VP), dorsolateral (DLP) and anterior (AP) prostatic lobes of mice under normal and high cholesterol diet in the various prostatic lobes. (B) Weight of each lobes were measured during necropsy and are represented as body weight indices (Prostate weight *vs.* body weight). Increased weight of VP in lxr-/- mice have been previously described (Viennois *et al*, 2012) (C) Histological morphology of dorsal prostate by Hematoxylin-Eosin staining. PCNA was detected by immunofluorescence in each genotype under high cholesterol diet. (D) *Cyclin D1* and *Cyclin D2* expression levels were analyzed by qPCR (N = 9/13 per group) in each genotype under normal and high cholesterol diet in dorsal prostatic lobes. * p<0.05, ** p<0.01 in Student's *t* test. Error bars represent the ± mean SEM.
(TIF)

Figure S2 Apoptosis Quantification in WT and *Lxr-/-* mice Fed Normal or High Cholesterol Diets. (A) TUNEL experiments on DLP from 5 months WT and *lxr-/-* mice fed a normal or high cholesterol diet for 5 weeks. Ep: Epithelium, St: Stroma (Scale bars = 50 µM). (B) Quantitative analysis of TUNEL experiments. Number of TUNEL positive cells per acini (N = 6). ** p<0.01 in Student's *t* test. Error bars represent the ± mean SEM.
(TIF)

Figure S3 Analysis of Microarray Datasets for WT or LXR Mutant Mice under Normal or High Cholesterol Diet. Two-colors 44K-whole mouse genome microarray datasets were analyzed using SpotFire Software. All gene expression profiles were plotted by Log ratio (*Y axis*) and Signal processed intensity (*X axis*) (green channel by default). Significant gene expression changes were determined by the threshold method with the following parameters: signal intensity (>250 processed signal), Log ratio (−0,3 <, >0,3) and *p*-value (<10^−7^). False positive hits were limited by filtrating the gene lists using dye swap datasets for each condition. This analysis resulted in the identification of 373 deregulated genes in array 1, 626 genes in array 2, 797 genes in array 3 and 1198 genes in array 4.
(TIF)

Figure S4 Ingenuity knowledge-based Pathway Analysis (IPA) for Canonical Pathways. The 463 genes list obtained from Venn analysis showed “Cancer, Organ Development, Cellular Growth and Proliferation” as the Top Network. Pathways analysis revealed in Top Bio Functions - Diseases and disorders that “Cancer” represented the second best *p*-value score with 146 genes associated.
(TIF)

Figure S5 Analysis of *Nkx3.1* and *Msmb* Expression in *Lxr*α, *Lxr*β Single Knockout Mice. *Nkx3.1* and *Msmb* expression levels were analyzed by qPCR (N = 9/13 per group) in each genotype under normal and high cholesterol diet in dorsal prostatic lobes. * p<0.05 in Student's *t* test. Error bars represent the ± mean SEM.
(TIF)

Figure S6 Analysis of *Uhrf1* expression. *Uhrf1* expression levels were analyzed by qPCR (N = 9/13 per group). * p<0.05 in Student's *t* test. Error bars represent the ± mean SEM.
(TIF)

Figure S7 Analysis of Inflammatory Status of Prostates (A) HE and IF against CD45 on the dorsal prostate lobe from lxr-/- mouse fed a high cholesterol diet. Spleen of a WT mouse was used as positive control. (B) RT-qPCR analysis of *Cd45* expression was performed with 5 month-old WT and *lxr-/-* mice under normal or high cholesterol conditions for 5 weeks (n = 9/13). Student's t-test: *P<0.05, **p<0.01, ***p<0.001. Error bars represent the ± mean SEM. (C) Hierarchical clustering of inflammatory genes compared between array 1 (+/+ normal *vs.* +/+ high chol.) and 4 (+/+ high chol. *vs.* lxr-/- high chol.) in order to identify specific gene signature. Genes have been clusterized in 3 groups.
(TIF)

Figure S8 Human Dataset analysis on normal gland, prostate carcinoma and metastsis. Oncomine heat maps and boxed plot analysis (http://www.oncomine.org) of *LXRβ*, *LXRα, EZH2, MSMB* and *NKX3.1* expression levels between healthy prostate glands, human PCa and metastasis in datasets referenced in [19] and [20] (n.s.; non-significant).
(TIF)

Text S1 Supporting Materials and Methods.
(DOCX)
