# Supplementary figures and images for: Liver X Receptors Protect from Development of Prostatic Intra-Epithelial Neoplasia in Mice
Source: PLoS Genet. 2013 May 9;9(5):e1003483. doi: 10.1371/journal.pgen.1003483 (PMC3649972; doi:10.1371/journal.pgen.1003483)

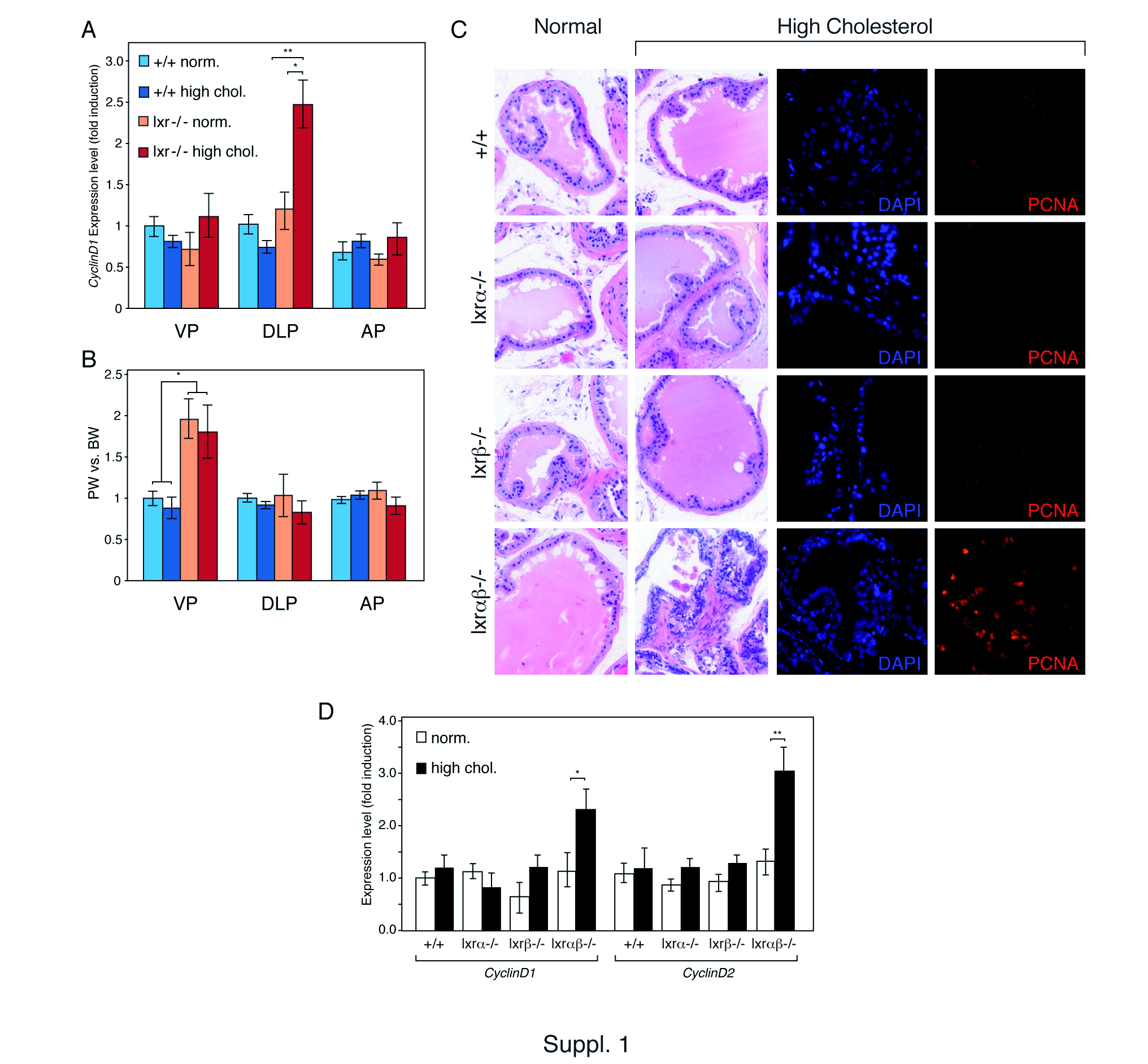

Supplement: Figure S1 — Analysis of Cell Proliferation in Lxrα, Lxrβ Single Knockout Mice and Weights of Prostatic Lobes. (A) CyclinD1 expression levels were analyzed by qPCR (N = 9/13 per group) in ventral (VP), dorsolateral (DLP) and anterior (AP) prostatic lobes of mice under normal and high cholesterol diet in the various prostatic lobes. (B) Weight of each lobes were measured during necropsy and are represented as body weight indices (Prostate weight vs. body weight). Increased weight of VP in lxr-/- mice have been previously described (Viennois et al, 2012) (C) Histological morphology of dorsal prostate by Hematoxylin-Eosin staining. PCNA was detected by immunofluorescence in each genotype under high cholesterol diet. (D) Cyclin D1 and Cyclin D2 expression levels were analyzed by qPCR (N = 9/13 per group) in each genotype under normal and high cholesterol diet in dorsal prostatic lobes. * p<0.05, ** p<0.01 in Student's t test. Error bars represent the ± mean SEM. (TIF) [file pgen.1003483.s004.tif]

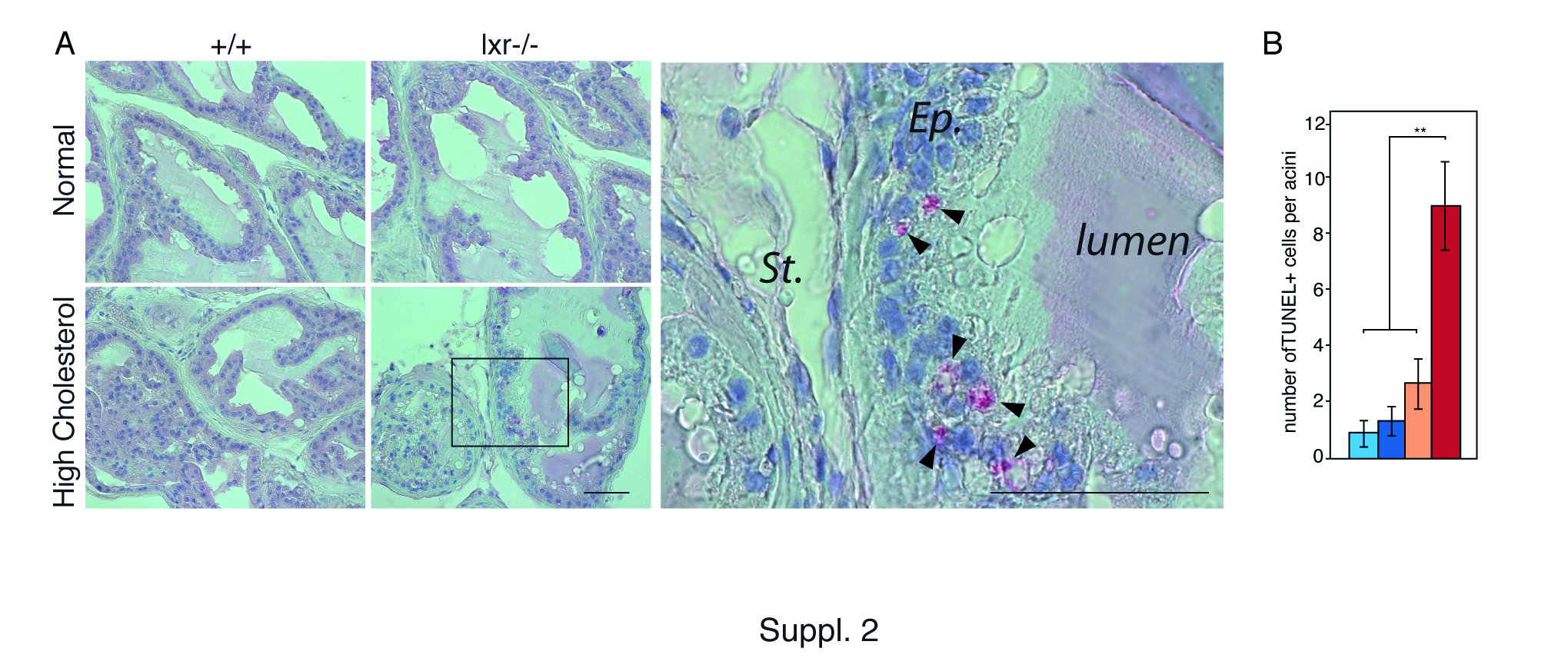

Supplement: Figure S2 — Apoptosis Quantification in WT and Lxr-/- mice Fed Normal or High Cholesterol Diets. (A) TUNEL experiments on DLP from 5 months WT and lxr-/- mice fed a normal or high cholesterol diet for 5 weeks. Ep: Epithelium, St: Stroma (Scale bars = 50 µM). (B) Quantitative analysis of TUNEL experiments. Number of TUNEL positive cells per acini (N = 6). ** p<0.01 in Student's t test. Error bars represent the ± mean SEM. (TIF) [file pgen.1003483.s005.tif]

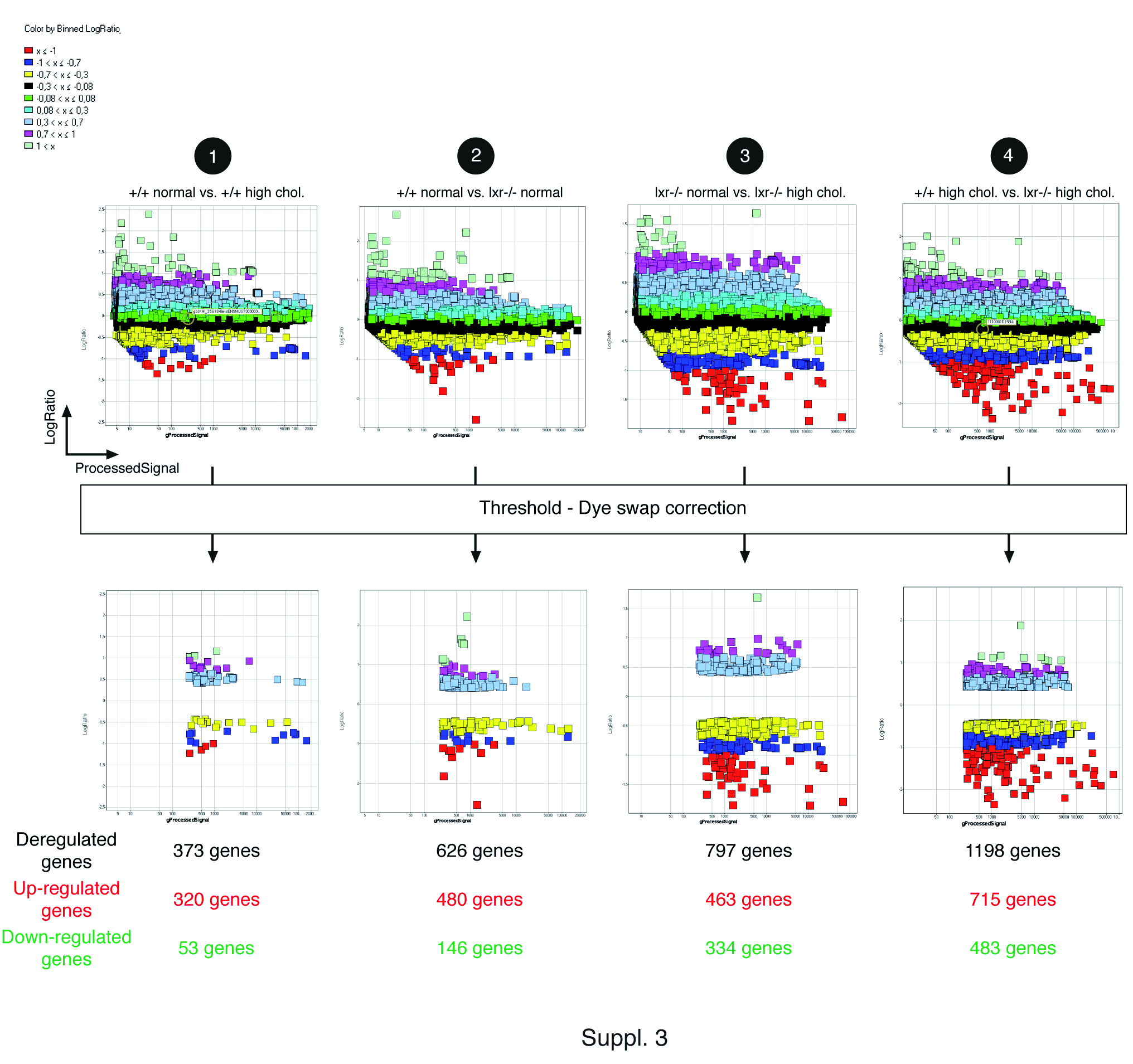

Supplement: Figure S3 — Analysis of Microarray Datasets for WT or LXR Mutant Mice under Normal or High Cholesterol Diet. Two-colors 44K-whole mouse genome microarray datasets were analyzed using SpotFire Software. All gene expression profiles were plotted by Log ratio (Y axis) and Signal processed intensity (X axis) (green channel by default). Significant gene expression changes were determined by the threshold method with the following parameters: signal intensity (>250 processed signal), Log ratio (−0,3 <, >0,3) and p-value (<10−7). False positive hits were limited by filtrating the gene lists using dye swap datasets for each condition. This analysis resulted in the identification of 373 deregulated genes in array 1, 626 genes in array 2, 797 genes in array 3 and 1198 genes in array 4. (TIF) [file pgen.1003483.s006.tif]

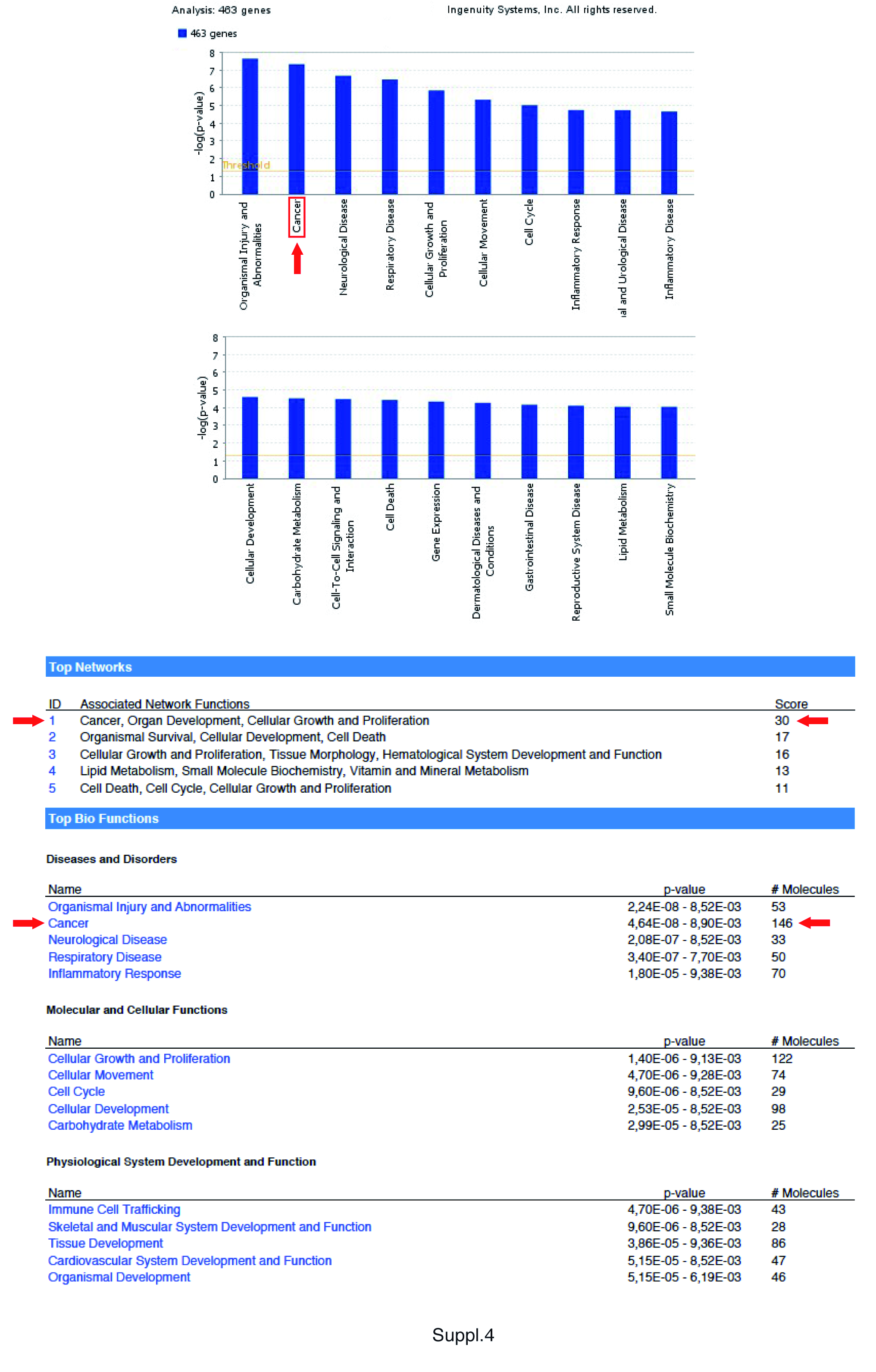

Supplement: Figure S4 — Ingenuity knowledge-based Pathway Analysis (IPA) for Canonical Pathways. The 463 genes list obtained from Venn analysis showed “Cancer, Organ Development, Cellular Growth and Proliferation” as the Top Network. Pathways analysis revealed in Top Bio Functions - Diseases and disorders that “Cancer” represented the second best p-value score with 146 genes associated. (TIF) [file pgen.1003483.s007.tif]

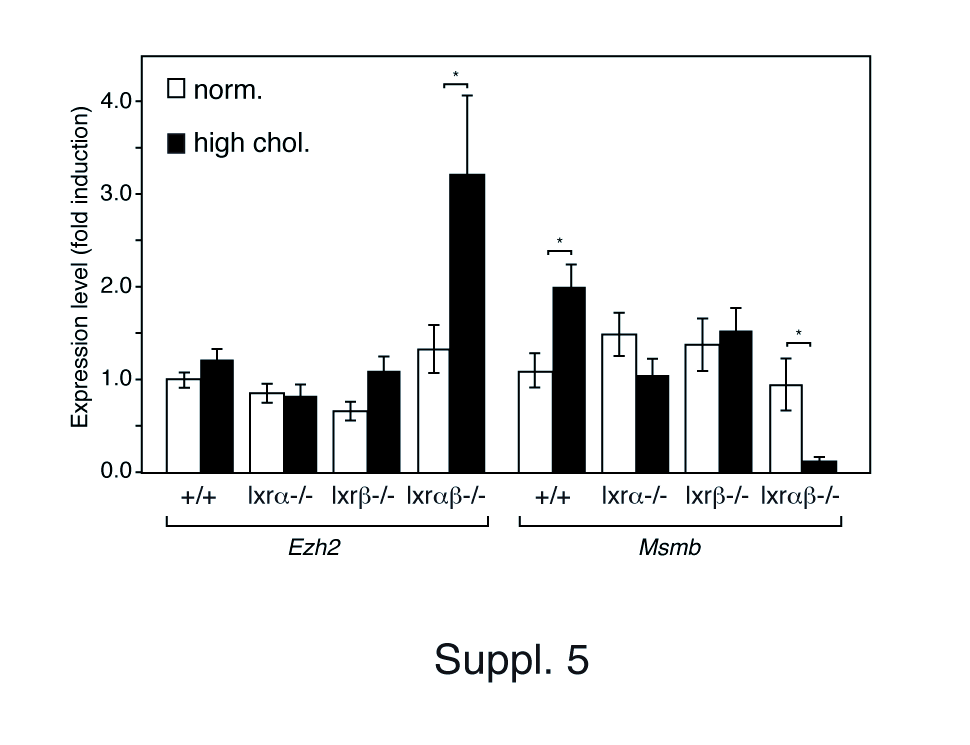

Supplement: Figure S5 — Analysis of Nkx3.1 and Msmb Expression in Lxrα, Lxrβ Single Knockout Mice. Nkx3.1 and Msmb expression levels were analyzed by qPCR (N = 9/13 per group) in each genotype under normal and high cholesterol diet in dorsal prostatic lobes. * p<0.05 in Student's t test. Error bars represent the ± mean SEM. (TIF) [file pgen.1003483.s008.tif]

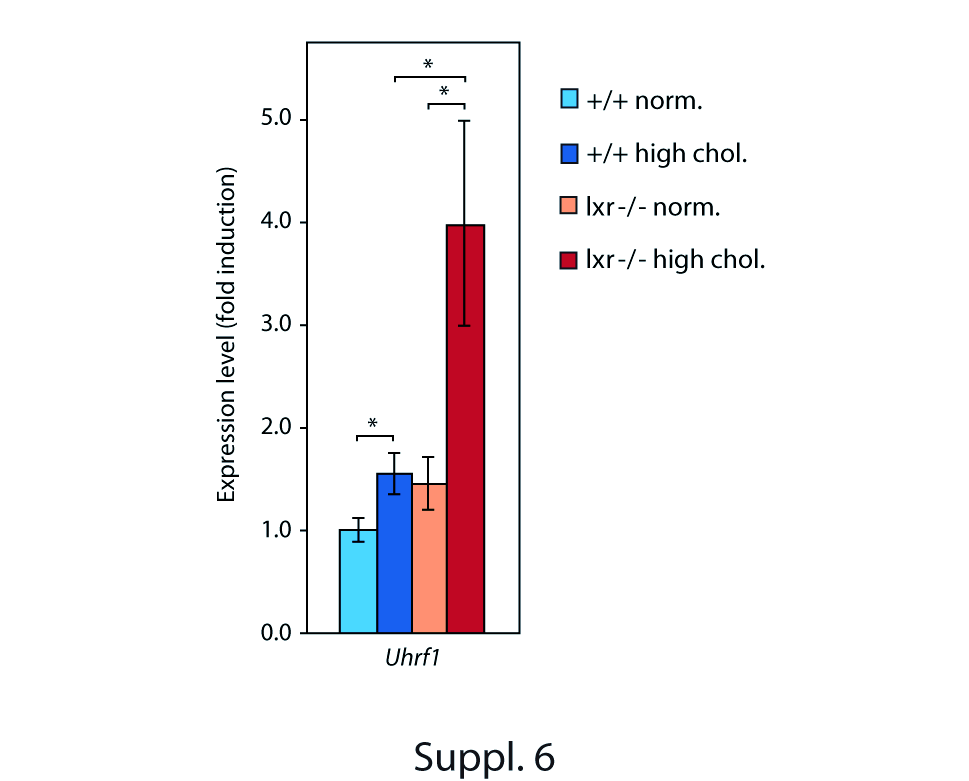

Supplement: Figure S6 — Analysis of Uhrf1 expression. Uhrf1 expression levels were analyzed by qPCR (N = 9/13 per group). * p<0.05 in Student's t test. Error bars represent the ± mean SEM. (TIF) [file pgen.1003483.s009.tif]

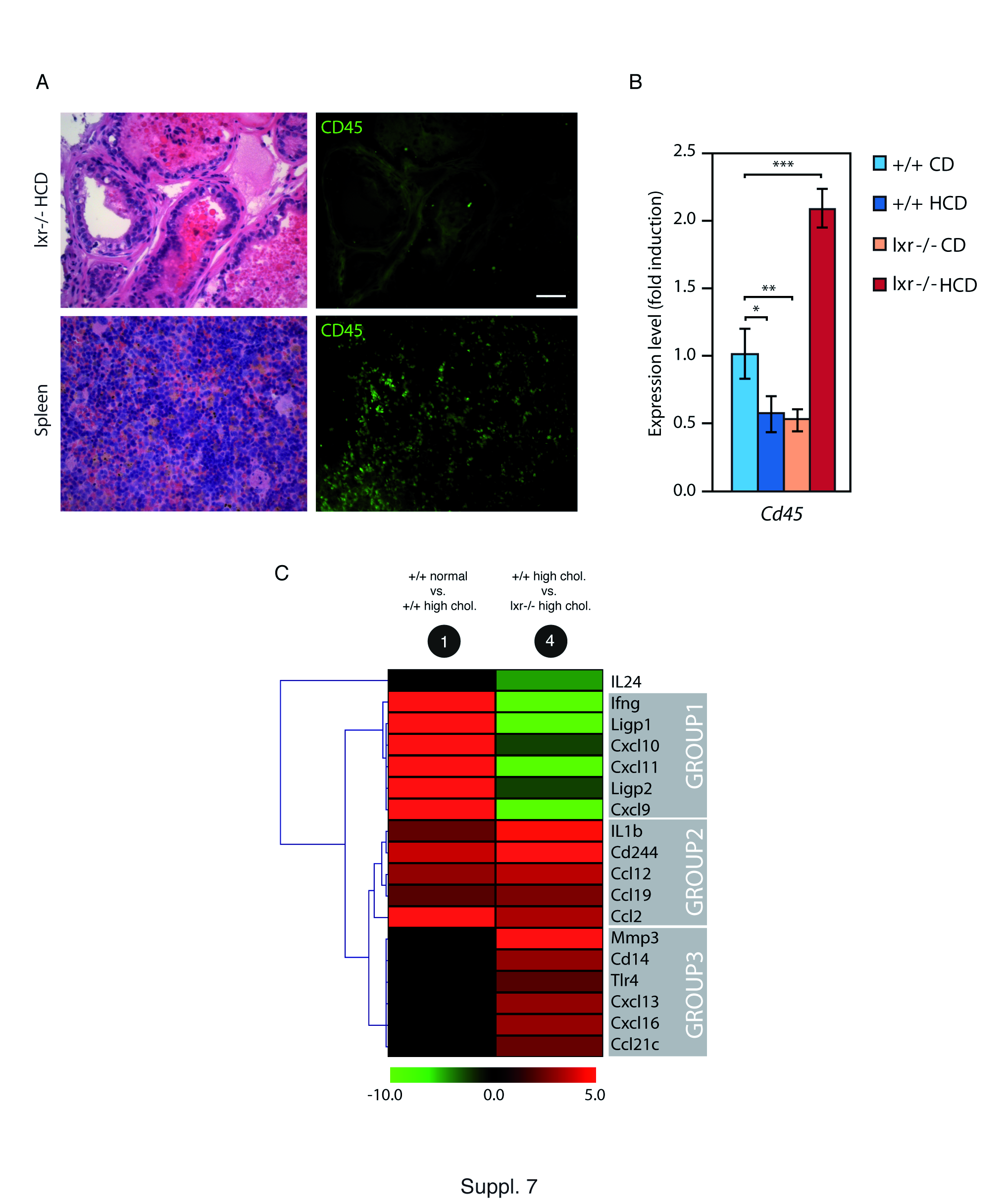

Supplement: Figure S7 — Analysis of Inflammatory Status of Prostates (A) HE and IF against CD45 on the dorsal prostate lobe from lxr-/- mouse fed a high cholesterol diet. Spleen of a WT mouse was used as positive control. (B) RT-qPCR analysis of Cd45 expression was performed with 5 month-old WT and lxr-/- mice under normal or high cholesterol conditions for 5 weeks (n = 9/13). Student's t-test: *P<0.05, **p<0.01, ***p<0.001. Error bars represent the ± mean SEM. (C) Hierarchical clustering of inflammatory genes compared between array 1 (+/+ normal vs. +/+ high chol.) and 4 (+/+ high chol. vs. lxr-/- high chol.) in order to identify specific gene signature. Genes have been clusterized in 3 groups. (TIF) [file pgen.1003483.s010.tif]

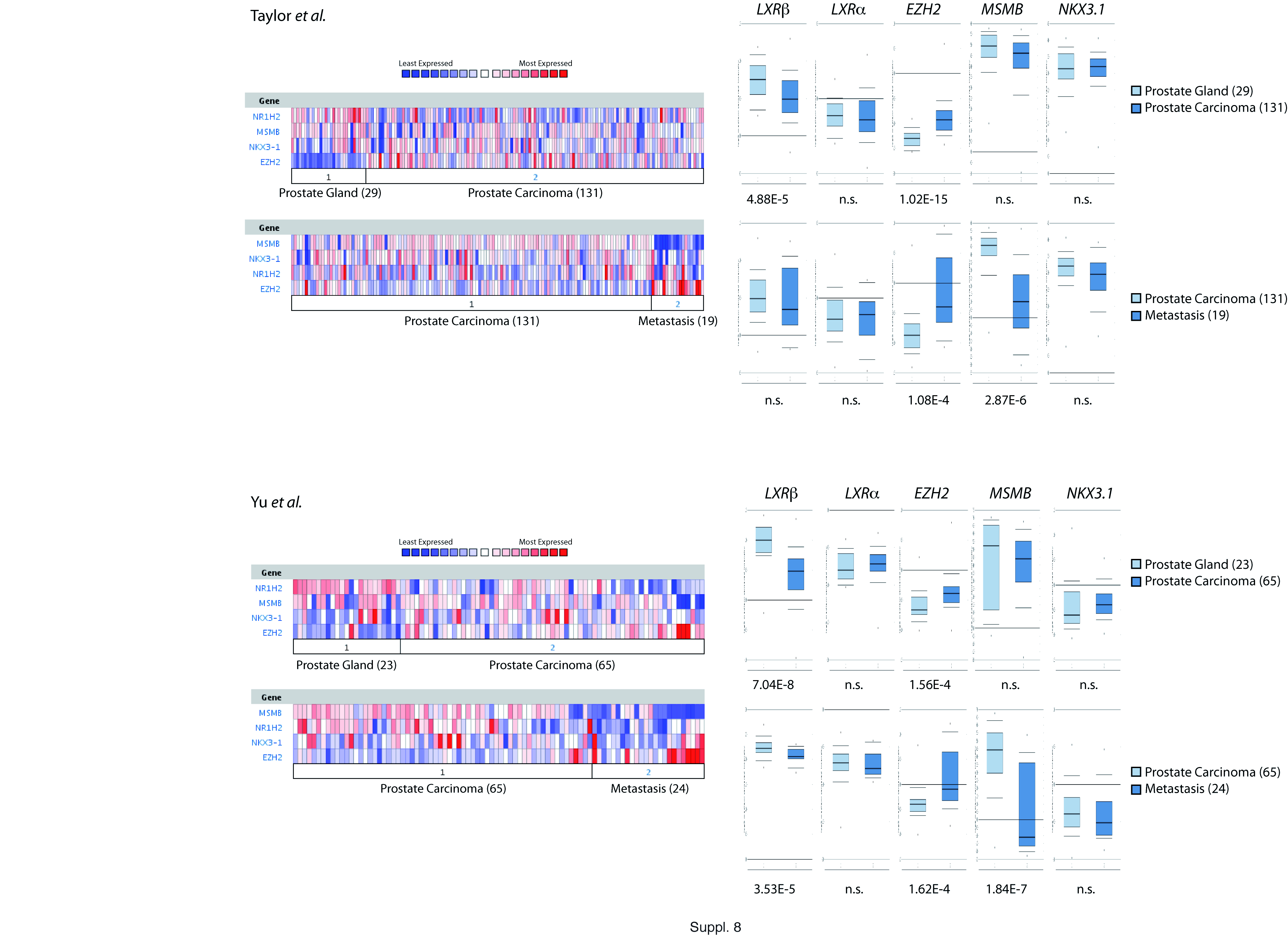

Supplement: Figure S8 — Human Dataset analysis on normal gland, prostate carcinoma and metastsis. Oncomine heat maps and boxed plot analysis (http://www.oncomine.org) of LXRβ, LXRα, EZH2, MSMB and NKX3.1 expression levels between healthy prostate glands, human PCa and metastasis in datasets referenced in [19] and [20] (n.s.; non-significant). (TIF) [file pgen.1003483.s011.tif]
