# Supplementary material for: Liver X Receptors Protect from Development of Prostatic Intra-Epithelial Neoplasia in Mice
Source: PLoS Genet. 2013 May 9;9(5):e1003483. doi: 10.1371/journal.pgen.1003483 (PMC3649972; doi:10.1371/journal.pgen.1003483)
Supplement: Text S1 — Supporting Materials and Methods. (DOCX) [file pgen.1003483.s012.docx]

**Text S1 - SI Materials and Methods**

**Animals.** *Lxrα-/-* and *Lxrβ-/-* knockout mice were housed and fed as described for *Lxrαβ*-/- and WT mice.

**Real-Time PCR.** qPCR analyses were performed as described in Material and Methods, Primer sequences were: mSREBF1fw GCCTGTACAGCGTGGCTGGG; mSREBF1rv TCTCCGTCAGCTGCCCCTGG; mIdolfw AGCGGCCTCTACCGAGCCAT; mIdolrv CGCCAAGTGGCCCTTCAGGT; mABCA1fw GGAGCTGGGAAGTCAACAAC; mABCA1rv ACATGCTCTCTTCCCGTCAG; mCycD1fw TCTCCTGCTACCGCACAAC mCycD1rv TTCCTCCACTTCCCCCTC; mCycD2fw TCCCGCAGTGTTCCTATTTC; mCycD2rv CTGGGGCTTCACAGAGTTGT; mEzh2fw CCTCTGAAGCAAATTCTCGG; mEzh2rv ACATCCTCAGTGGGAACAGG; mNkx3.1fw GGAGGACCCACCAAGTATCC; mNkx3.1rv ATGGCTGAACTTCCTCTCCA; mMsmbfw AGTCCTGCTTTGCCACCATG; mMsmbrv CGTGCAGTTCTTCTTCCAGGGAGT; m18Sfw GGGAGCCTGAGAAACGGC; m18Srv GGGTCGGGAGTGGGTAATTT; mFasfw CCCCAACCCTGAGATCCCA mFasrv TTGATGCCCACGTTGCC; mScd2fw TTCCTCATCATTGCCAACACC mScd2rv GGGCCCATTCATACACGTCA; m36b4fw GTCACTGTGCCAGCTCAGAA m36b4rv TCAATGGTGCCTCTGGAGAT; mUhrf1fw ACGGTGCCTACTCATTGGTC; mUhrf1rv GCTTCTGGTCAGAGGACTGG; *Cd45* expression was analyzed using Taqman Genes Expression assays probes (Applied Biosystem, Villebon-sur-Yvette). qPCR results were normalized alternatively using *36b4* or *18S* as a standard.

**Transcriptomic and Pathway Analyses.** Total RNAs of the dorsal prostates of 7 month-old WT and LXR null mice fed a normal or hypercholesterolemic diet were isolated using NucleoSpin® RNA II column kit (Macherey-Nagel, Hoerd, France). The quantity of each total RNA sample was determined by spectrophotometry and the size distribution was assessed using an Agilent Bioanalyzer (Agilent, Wilmington, DE). For each group of mice, 3 mRNA samples were pooled and converted into labeled cDNA using fluorescent nucleotides coupled with either Cy3 or Cy5 and the Low DNA Input Linear Amplification Kit (Agilent Technologies, Palo Alto, CA), following manufacturer’s protocol. cDNA quality was assesed using an Agilent Bioanalyzer. Equal amounts of Cy3 and Cy5-labeled cDNA from two different samples were hybridized to Agilent 44K Whole Mouse Genome microarrays and dye swaps were performed to exclude false positive/negative. The hybridized array was then washed and scanned and data were extracted from the scanned image using Feature Extraction version 9.5 (Agilent Technologies). For each microarray, log ratio, fold-change and *p*-value were determined using the Rosetta Resolver Gene Expression Analysis System. Microarrays were deposited in the EBI MIAME-compliant database (E-MTAB-546). Microarray datasets were analyzed using Spotfire software (TIBCO, Palo Alto, CA), Ingenuity Pathway Analysis (IPA 8.7 – 3203) (Ingenuity systems, Redwood City, CA) and Multi Experiment Viewer software (MeV_4_7 Version 10.2) (<http://www.tm4.org/mev>) [44].

**Chromatin Immunoprecipitation.** qPCR was performed with the following primers : mNKX3.1(I)fw GTTCCCTGCAGAGCAGATTC; mNKX3.1(I)rv GGAAACCGAGGAAAGCCTAC; mNKX3.1(II)fw TCTTAAGAATACTCTTTCCTGGTGTT; mNKX3.1(II)rv TCGTTTACTGAGTTGACCAGG; mNKX3.1(III)fw ACACACATCCAGGATCCACA; mNKX3.1(III)rv ACCCTGAAGAAGTGAGCAGG; mMmsb(I)fw TTTCTGGCTGCTTGTTTCCT; mMmsb(I)rv CCGTCTGCACTCCTGAAAGT mMmsb(II)fw CATACGGCTGAAGACCCACT; mMmsb(II)rv TGTCTCCTGCCTTCCTCTGT; mMmsb(III)fw GTACACCAACGTGGCTCCTT; mMmsb(III)rv AACCATGGATTCAATTCCCA.

**Reference**

46. Saeed AI, Sharov V, White J, Li J, Liang W, et al. (2003) TM4: a free, open-source system for microarray data management and analysis. Biotechniques 34: 374–378.
